# *In operando* imaging of self-catalyzed formaldehyde burst in methanol oxidation reactions under open circuit conditions†
†Electronic supplementary information (ESI) available: Experimental details, data analysis, and descriptions of movies. See DOI: 10.1039/c7sc05347a


**DOI:** 10.1039/c7sc05347a

**Published:** 2018-02-26

**Authors:** Liang Yuan, Meng Li, Tinglian Yuan, Yimin Fang, Wei Wang

**Affiliations:** a State Key Laboratory of Analytical Chemistry for Life Science , School of Chemistry and Chemical Engineering , Nanjing University , Nanjing 210093 , China . Email: wei.wang@nju.edu.cn

## Abstract

We employ a surface plasmon resonance imaging (SPRi) technique to monitor the *in operando* process of formaldehyde (HCHO) production during methanol oxidation with high spatial and temporal resolutions. While common wisdom suggests HCHO is generated as an intermediate during continuous electron transfer towards CO_2_, we find that the majority of HCHO is produced *via* self-catalyzed chemical and electrochemical reactions under open-circuit conditions, which lead to an unprecedented HCHO burst immediately after withdrawal of external potential. Because open-circuit conditions better represent the operating environments of practical direct methanol fuel cells (DMFCs), this work uncovers a hidden pathway of HCHO accumulation by adopting a quantitative and *in operando* SPRi technique for the first time. These theoretical and technical advances are anticipated to help the fundamental understanding of the comprehensive mechanism of methanol oxidation with implications for improving the performance of DMFCs.

## Abstract 2

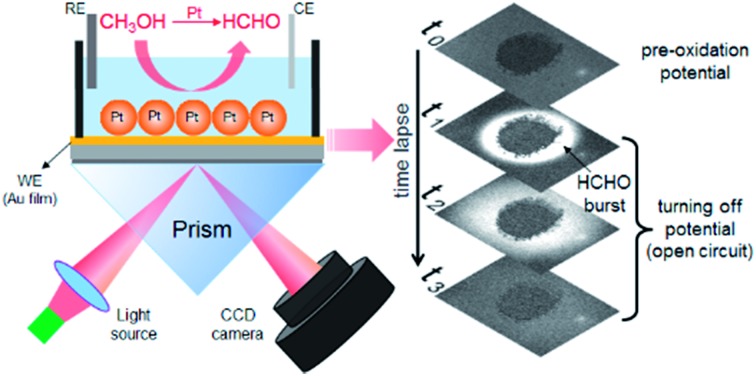
A wave-like HCHO burst is *in situ* observed during electro-oxidation of methanol on Pt under open circuit conditions by SPR imaging.

## Introduction

Despite the theoretically promising future held by DMFCs in portable devices and electric vehicles, their commercialization has been largely hampered by some technical challenges.^1^,^2^ One of the major concerns is the insufficient cell voltage and current density due to the formation of undesired byproducts such as HCHO *via* a 2-electron process.^3^–^5^ However, the detailed mechanism of HCHO production in DMFCs remains under debate because it has proven difficult to detect HCHO with existing *in situ* spectro-electrochemical techniques such as infrared spectroscopy and mass spectrometry due to the interference of other species in solution.^4^,^6^–^9^ And they can only determine the final concentration of HCHO after reaction completion.^3^,^10^ This revealed that HCHO was the major byproduct of methanol oxidation on Pt catalysts, especially when operating at a lower cell potential.^3^,^5^,^10^ So an *in operando* capability to detect HCHO as a function of time (or potential) is highly desired to uncover the long-standing mystery of HCHO formation in DMFCs.

SPRi is a wide-field optical imaging technique that utilizes the planar SPR effect to map the distribution of the refractive index with sub-micron spatial and sub-millisecond temporal resolutions.^11^–^13^ Since its invention in the 1990s, SPRi has become a powerful and popular technique for determining the binding kinetics between biomolecules and their ligands in protein and DNA microarrays.^14^,^15^ In addition to biological applications, Tao's group recently found that, when triggering electrochemical reactions on a gold substrate and monitoring the time-lapse SPRi images at the same time, a map of electrochemical current could be obtained because reaction products often exhibited different refractive indices to reactants.^16^–^18^ Inspired by this principle, the capability of SPRi to investigate electrochemical reactions of individual electroactive nanoparticles has been demonstrated by others and us.^19^–^22^ However, the utilization of SPRi to study methanol oxidation, an important anode reaction in DMFCs, has not been demonstrated yet.

In the present work, we employed a SPRi technique to monitor the production of HCHO during electro-oxidation of methanol on Pt catalysts in a quantitative and *in operando* manner. Because the refractive index of HCHO (product) is much higher than that of CH_3_OH (reactant), the electrochemical reactions lead to increased optical signals in the time-lapse SPRi images. Temporal and spatial resolutions of SPRi allow for studying the reaction kinetics and spatial distribution of this important byproduct HCHO, respectively. While common wisdom suggests that HCHO would be produced as an intermediate during the continuous electron transfer towards CO_2_, our results revealed an unexpected HCHO burst which rapidly diffused from the Pt catalyst to surrounding medium immediately after the withdrawal of external potential (*i.e.*, under OCP conditions). Systematical investigations attributed this phenomenon to the rapid chemical and electrochemical oxidation of methanol by surface-adsorbed oxidative species on Pt catalysts. A self-catalyzed production of HCHO was further detected by analyzing the corresponding kinetic data, which underscored the quantitative and *in operando* features of the SPRi technique.

## Results and discussion

### HCHO burst phenomenon under open circuit conditions

**Fig. 1:**
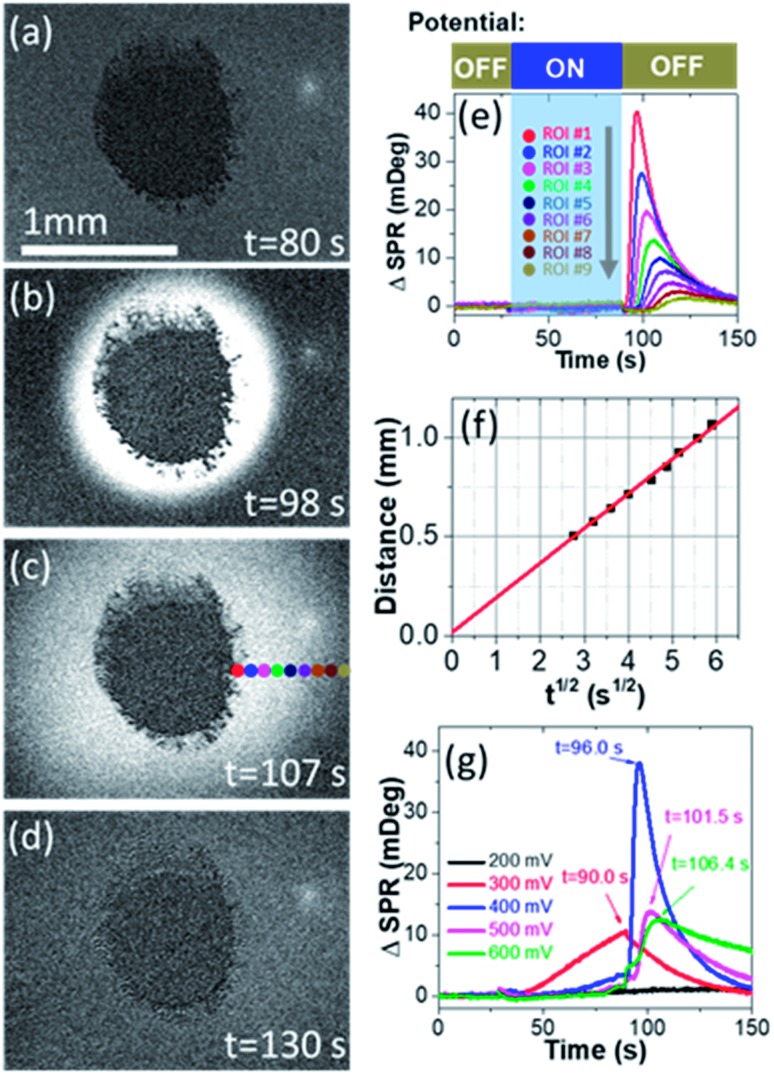
(a)–(d) Time-lapse SPR images of the Pt-disk in EL-1 solution during methanol oxidation at different moments of (a) 80, (b) 98, (c) 107 and (d) 130 s, respectively. (e) SPR intensity curves of nine small ROIs adjacent to the Pt-disk *vs.* time during the potentiostatic measurement. The locations of these ROIs are indicated by colored dots in (c). (f) The distance of ROIs to the center of the Pt-disk as a function of the time required for the ROI to reach the local maximal concentration (*t*_max_). (g) SPR intensity curves of ROI #1 when applying different pre-oxidation potentials (from 0.2 to 0.6 V).

We first prove the electrocatalytic activity of Pt nanoparticles (PtNPs) for efficient methanol oxidation. PtNPs are deposited on a gold-coated coverslip to form a porous and disk-like Pt-catalyst (Pt-disk), which can efficiently catalyze the electrochemical oxidation of methanol (Fig. S1 and S2†). This Pt-disk chip is subsequently placed on top of a prism for SPRi experiments. A representative SPRi image is shown in Fig. 1a. The round-shaped dark-pattern in the center represents the Pt-disk because the reflectivity from the thick Pt-layer is much smaller than that of the surrounding solution where the total internal reflection condition remains valid. Any increase (decrease) in the local refractive index will quantitatively increase (decrease) the intensity in the corresponding region-of-interest (ROI) in SPRi images.

A diffusive burst is observed when watching the time-lapse SPRi images during the methanol oxidation experiments whose procedure is described as follows. The SPRi images are continuously recorded at a speed of 7.6 fps for 30 seconds in the absence of an external potential to acquire a baseline. Subsequently, a pre-oxidation potential of +500 mV *vs.* NHE is applied to the Pt-disk in the presence of electrolyte (0.5 M H_2_SO_4_ + 1.0 M CH_3_OH, defined as EL-1) and kept for another 60 seconds. During this period of time, SPRi images (Fig. 1a) remain unchanged after removing the charging background which is uniform all over the gold substrate. At the 90th second, the pre-oxidation potential is turned off again (*i.e.*, open-circuit conditions). Surprisingly, the solution surrounding the Pt-disk undergoes a wave-like burst in the recorded SPRi images (Fig. 1b–d and Movie S1†), clearly indicating a massive release (production and diffusion) of some chemical species with a higher refractive index.

Quantitative analysis confirmed the diffusive nature of this burst. Fig. 1e shows the SPR intensity curves of nine adjacent ROIs, whose locations are indicated by colored dots in Fig. 1c. The kinetic features of these curves are well consistent with the 3-dimensional semi-infinite diffusion model. When assuming that *M* molecules are released from a point-source in the substrate–solution interface at time zero and subsequently diffuse into the solution, species concentration *C*(*r*, *t*) as a function of radial distance (*r*) and time (*t*) can be resolved using the following equation:
1

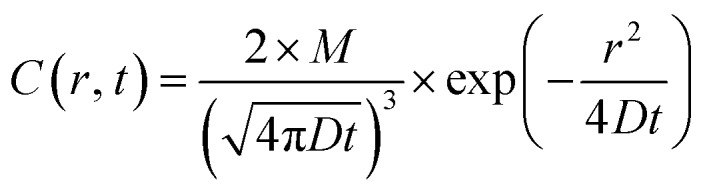

where *D* is the diffusion coefficient. For each ROI, the initial concentration before diffusion is zero. Subsequently, the local concentration goes up when species are diffusing over this area. Since the species amount is limited, the local concentration would eventually decrease towards zero again. Because of the mm-scale size of Pt-dots, it is not suitable to directly apply this simplified equation involving the point-source. Instead, the diffusion distance (*L*) was calculated by adding the radial diffusion length from the edge of the Pt-dot to its radius (*r* = 0.4 mm). The diffusion model predicts that, for each ROI, the time required to reach the maximal concentration (*t*_max_) is a function of its distance (*L*) to the center of the Pt-disk (
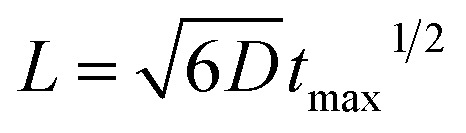
, ESI Section 2.2†). As shown in Fig. 1f, the experimental results indeed follow this relationship. The diffusion coefficient is thus determined to be 4.8 × 10^–9^ m^2^ s^–1^, which is in the reasonable range of diffusion coefficients of small molecules in water.

We further examine the dependence of HCHO burst on the pre-oxidation potential (Fig. 1g). When the pre-oxidation potential is lower than 200 mV, no HCHO burst is observed at all (black curve). When applying a potential of 300 mV, gradual release of HCHO appears in the potential ON stage, which gradually decreases after the withdrawal of potential. The most significant HCHO burst is observed when applying a potential of 400 mV. Further increasing the potential is also able to trigger the HCHO burst, but with an obvious delay in *t*_max_ for the same ROI (Fig. S3†), which will be discussed in the last section. These results demonstrate that HCHO burst is a consequence of the electrochemical reactions in the pre-oxidation stage, and it is triggered by the withdrawal of potential. Note that HCHO is continuously produced in the pre-oxidation stage when applying a higher potential (*E* > 0.4 V). However, if a high potential is held, the HCHO is released at a slower rate due to the effective production of CO_2_ (Fig. 1g blue, magenta and green curves).

### Identification of HCHO as the chemical species during burst

**Fig. 2:**
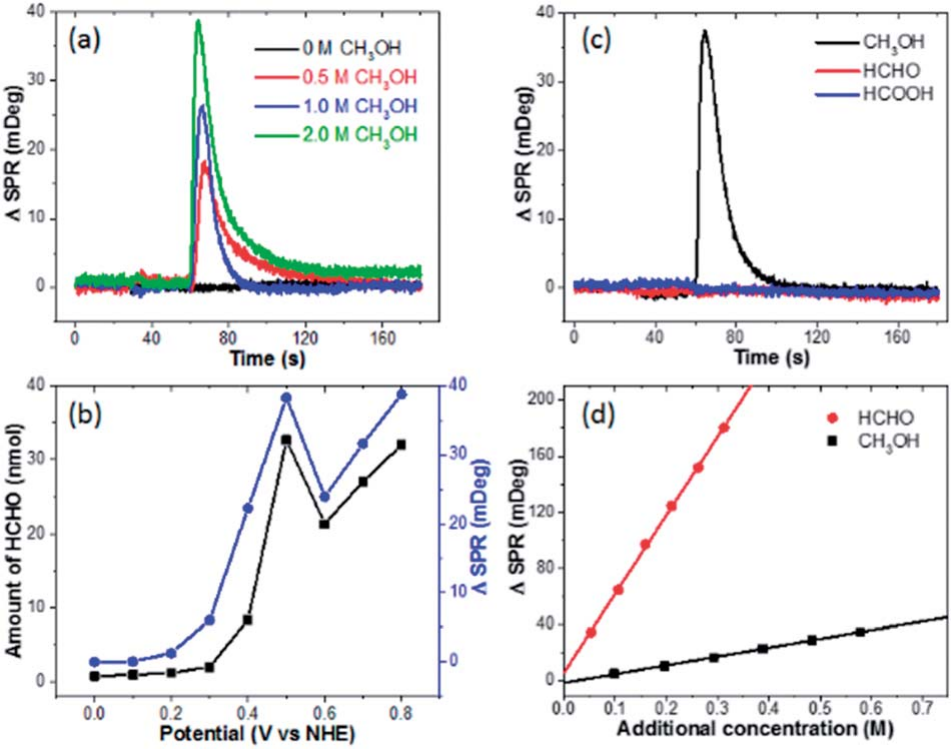
SPR intensities of the Pt-disk chip in 0.5 M H_2_SO_4_ electrolyte (EL-2) mixed with (a) different concentrations of methanol or mixed with (c) different reactants including 1.0 M methanol, formaldehyde and formic acid, respectively, during the potentiostatic measurement, in which all the applied pre-oxidation potentials are 0.5 V for 30 s. (b) Comparison of generated HCHO concentrations (black line) and maximal SPR intensities (blue line) as a function of the applied pre-oxidation potential. The end-point HCHO concentrations are determined from the spectrometric measurements. (d) SPR intensity changes as a function of additional concentration of CH_3_OH and HCHO.

We subsequently clarify the chemical identification of the burst. First, the optical signal is proportional to the CH_3_OH concentration, and no burst is observed in the absence of methanol (Fig. 2a). These results together with the dependence on pre-oxidation potential (Fig. 1g) indicate that the electro-oxidation products of methanol are responsible for the burst. Second, a well-established spectrometric assay (see Fig. S4† for details) is utilized to determine the end-point concentration of HCHO at different pre-oxidation potentials, which shows excellent agreement with the optical measurements (Fig. 2b).^23^,^24^ It should be noted that the maximal SPR intensity was calculated by averaging the SPR intensity of a ring-shaped region-of-interest surrounding the Pt-disk (Fig. S5†). Third, when performing the same experiments by replacing CH_3_OH with HCHO or HCOOH, no burst is observed at all (Fig. 2c). These results indicate the role of the 2-electron oxidation product HCHO. In addition, optical calibration curves (Fig. 2d) and the experimentally determined refractive index values (Fig. S6†) obtained by adding different solutions to EL-1 (0.5 M H_2_SO_4_ + 1.0 M CH_3_OH) show that the refractive index of HCHO solution is much larger than that of CH_3_OH solution with the same molar concentration. This means that when converting CH_3_OH to HCHO, the SPR intensity would increase. The increased refractive index due to the added H_2_SO_4_ solution is also displayed and the influence of adding HCHO is about 5 times larger than that of adding H_2_SO_4_ (Fig. S6†). During the oxidation of CH_3_OH, protons and HCHO are concomitantly generated and both will change the refractive index. It is difficult to differentiate the contributions from each other, and the SPR signal is actually contributed by both HCHO molecules and protons.^18^ According to the dependence between the HCHO burst and pre-oxidation potential (Fig. 1g) as well as the consistence between final HCHO concentration and optical signal intensity (Fig. 2b), the generated HCHO molecules play critical roles in the obtained SPR signal of the burst.

Many literature reports using *ex situ* techniques have also shown that HCHO is one of the major byproducts of methanol electro-oxidation on Pt electrodes. Systematic studies by Korzeniewski's group using fluorometric assays revealed that the HCHO yield was firstly increasing with electrode potential, reaching the maximum at 500 mV.^3^ A higher electrode potential would facilitate the 4-electron and 6-electron processes to produce HCOOH or CO_2_, leading to decreased HCHO yield. Such potential dependence is in good agreement with our results. Chromatography analysis conducted by Iwasita's group also confirmed these conclusions.^5^ In a recent work by Chen, HCHO was attributed to the major product when mixing methanol with a pre-oxidized Pt electrode under open circuit conditions.^25^ Therefore, we conclude that the rapid production and diffusion of HCHO on the Pt-disk immediately after the potential withdrawal is responsible for the burst phenomenon.

### Reaction mechanisms of HCHO burst under open circuit conditions

**Fig. 3:**
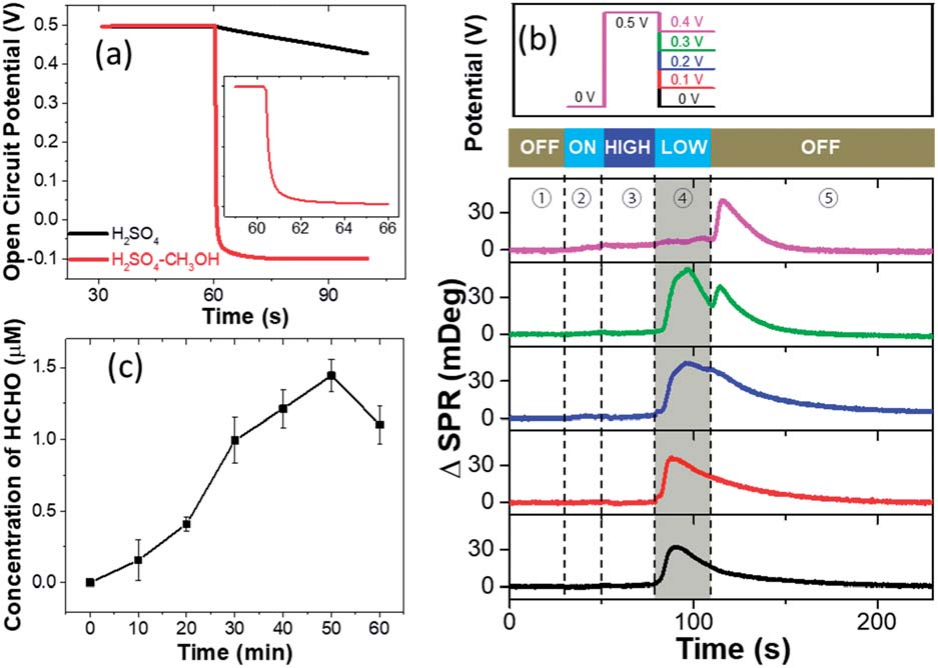
(a) Time evolution of the open circuit potential at the Pt-disk chip in EL-1 and EL-2 after holding the potential at a pre-oxidation potential of 0.5 V for 30 s and subsequent switching off the applied potential control at 60 s together with recording the OCP variation. Inset shows the magnified view of the moment of abrupt OCP change. (b) The SPR intensity of the Pt-disk chip in EL-1 from 0.5 V to 0 V, 0.1 V, 0.2 V, 0.3 V, and 0.4 V, respectively. The curve is composed of five stages: no potential for 30 s; quiet time at 0 V for 20 s; 0.5 V applied as a pre-oxidation potential for 30 s; steps to different lower potentials for another 30 s; turning off the potential for 120 s. (c) The concentration of yielded HCHO from the experiment of pumping CO gas into EL-1 solution without potential control, which is detected by the spectrometric measurement.

We next discuss the reaction mechanisms of HCHO burst after the withdrawal of potential. It is known that electrochemical reactions under OCP conditions still take place in a similar way as it is driven by external potentials.^25^ The major difference is that the driving potential under OCP conditions changes over time during the reaction process. Normally in pure H_2_SO_4_ solution, the OCP declines slowly and reverts to the original equilibrium state (Fig. S7†). Interestingly, it is found that the OCP rapidly decreases from 0.5 V to *ca.* –0.1 V within 5 seconds in the presence of CH_3_OH (Fig. 3a). OCP drops of the pre-oxidized Pt electrode in the presence of methanol have been frequently reported as an indicator of electron injection from solution to the electrode due to the methanol oxidation.^25^,^26^

A controlled potential experiment is also conducted in order to measure the amount of injected electrons during the HCHO burst. Instead of directly turning off the potential after 30 s pre-treatment at 0.5 V, different potentials are applied to the Pt-disk for another 30 s before turning off (upper panel in Fig. 3b). The corresponding SPR intensity curves from the Pt-disk are shown in the lower panel. When a 0.4 V potential is applied, HCHO burst would not appear until the potential withdrawal at the 120th s (magenta curve). It demonstrates that the OCP needs to be lower than 0.4 V to massively produce HCHO. The application of 0.3 V potential results in HCHO burst in the first 30 seconds. Another small peak shows up after the potential withdrawal (green curve), suggesting further production of HCHO at an OCP lower than 0.3 V. A similar but smaller secondary peak is also observed for 0.2 V (blue curve). An electrode potential of 0.1 V (red curve) or 0 V (black curve) immediately leads to the HCHO burst and no further peak is detected when turning off the potentiostat at the 120th second. These results indicate that the HCHO burst is a consequence of reduced interfacial potential as long as it is lower than 0.3 V. A lower potential leads to a faster HCHO production rate in the range from 0.3 to 0 V. This potential can be provided by either an OCP or a potentiostat. An oxidative current can be obtained by differentiating the chronoamperometric current of stage 4 in the presence and absence of reactant methanol, further indicating the electron injection caused by methanol oxidation on the Pt-disk under open-circuit conditions (Fig. S8†). The total amount of charge injected (*Q*) is calculated to be 1.5 mC (14 nmol electrons) by integrating the above differential current over time.

On the other hand, the amount of HCHO produced in one burst is calculated by two independent methods. In the first approach, we determine the final concentration of HCHO in the solution by using a spectrometric assay. The results reveal that 69.3 μM (42 nmol) HCHO is produced in one burst. The second approach utilized the diffusion model to calculate the amount of HCHO (*M*) from the time-dependent concentration evolution (Fig. 1e). Analysis of Fig. 1e gives us a total HCHO amount of 48 nmol. The consistency between the two methods not only demonstrates the validity of both approaches, but also suggests that the observed HCHO burst contributed the majority of the HCHO molecules generated during the entire experiments. In other words, the *in operando* results in the present work prove that the majority of the HCHO byproduct is produced under the open-circuit conditions, rather than being generated as an intermediate species during continuous electron transfer processes towards CO_2_.

**Fig. 4:**
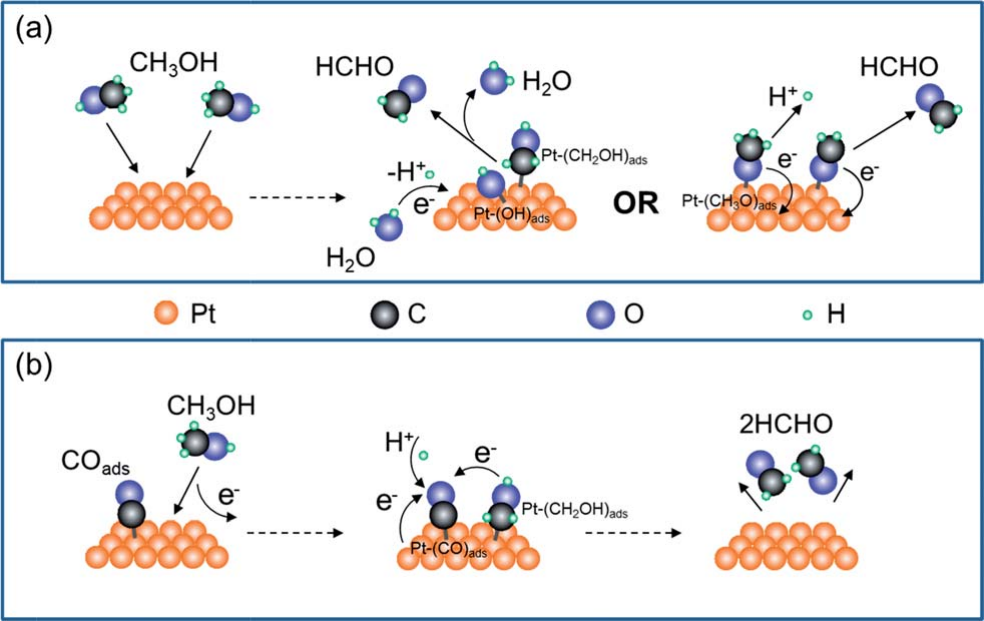
Illustration of the mechanism of methanol oxidation on the Pt-disk chip. (a) The electrochemical pathway for generating HCHO with both hydroxymethyl (CH_2_OH) and methoxide (CH_3_O) formation as the first step, respectively. (b) The chemical pathway for producing HCHO and diffusing from the Pt-disk surface when switching off the potential control or at comparatively lower potentials.

Production of HCHO follows both electrochemical and chemical pathways (Fig. 4). Electrochemically produced HCHO molecules should be *Q*/2 (7 nmol) because it is a 2-electron process, which is six times less than the total HCHO amount (*M*). The difference must be contributed by chemical reactions between methanol and pre-oxidized species on the Pt-disk. Previous spectrometric evidence has shown that Pt–C=O is the major oxidative species in this potential range (0.5 V). Therefore, we propose the following electrochemical [eqn (2)] and chemical [eqn (3)–(5)] pathways:
2

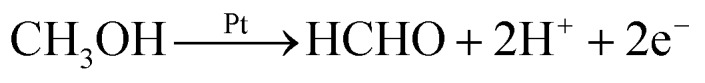

3 Pt + CH_3_OH → Pt–(CH_2_OH)_ads_ + H^+^ + e^–^

4 Pt–CO_ads_ + Pt–(CH_2_OH)_ads_ + H^+^ + e^–^ → 2HCHO + 2Pt

or,

5 Pt–CO_ads_ + CH_3_OH → 2HCHO + Pt

The chemical process is enabled by surface-adsorbed oxidative species Pt–CO_ads_. Although a certain interfacial potential is still required, the net interfacial electron transfer is zero [eqn (5)].

In order to validate the capability of Pt–CO_ads_ species to oxidize CH_3_OH chemically, another chemical oxidation experiment is also conducted in the absence of potential control. We continuously blow CO gas (bubbles) into the acidic methanol electrolyte, and detect the concentration of HCHO in the solution with the spectrometric assay. We clearly observed the production of HCHO after bubbling CO for 15 minutes (Fig. 3c). The HCHO concentration gradually increases while extending the reaction time. The abnormal decrease of HCHO production after 60 minutes may be a result of the escape of HCHO molecules from the solution due to long-term gas flow. These results demonstrate that Pt–CO_ads_ species is able to oxidize the methanol molecules in the solution without an external potential, thus supporting the chemical pathway proposed above.

### Self-catalyzed methanol oxidation

The methanol oxidation under open-circuit conditions exhibits an obvious feature of self-catalysis.^27^–^29^ After potential withdrawal, it often takes several seconds before the appearance of HCHO burst as shown in Fig. 1g. A longer delay is observed at higher pre-oxidation potentials (Fig. S3†). That's because both chemical and electrochemical oxidation of CH_3_OH required fresh metallic Pt atoms to enable the dissociative adsorption of CH_3_OH molecules [eqn (2)].^30^ However, most of the surface Pt atoms have already been oxidized to form Pt–CO_ads_ at high pre-oxidation potentials. Therefore, after potential withdrawal, one un-occupied Pt atom (with rather low chance) adsorbs one CH_3_OH molecule and reacts with the adjacent Pt–CO_ads_ to produce two HCHO molecules and to release two fresh Pt atoms [eqn (5)].^31^ This chain-like process increases the number of un-occupied Pt atoms, and further accelerates the HCHO process *via* a self-catalysis mechanism, until all the Pt–CO_ads_ species are exhausted. Higher pre-oxidation potentials will cause less un-occupied Pt atoms in the beginning, and thus requires a longer time to initiate the HCHO burst (Fig. 1g). This hypothesis is also supported by the absence of HCHO burst at a pre-oxidation potential of 0.3 V (red curve in Fig. 1g). Because this potential is not high enough to convert all Pt–CH_2_OH to Pt–CO_ads_, these two species co-exist all the time. Therefore, no ignition process is required to produce HCHO effectively. Instead, a monotonic decrease in the HCHO production rate is observed with no time delay. According to the diffusion theory, the observation of a HCHO burst requires the rapid formation and release of HCHO molecules within a short time. Otherwise, slow production would broaden and weaken the peaks in SPR intensity curves (red curve in Fig. 1g).

## Conclusions

In summary, we have demonstrated the capability of an *in operando* SPRi technique to study the dynamic production of HCHO during methanol oxidation for the first time, leading to the discovery of an unexpected HCHO burst phenomenon under open circuit conditions. Self-catalyzed electrochemical and chemical oxidation of methanol by the oxidative Pt–CO_ads_ species is responsible for the rapid production and subsequent diffusion of HCHO molecules, accompanying the observed HCHO burst. The present work provides both theoretical and technical advances in understanding the detailed reaction pathways in DMFCs. From a theoretical point of view, most of the methanol electro-oxidation studies have been conducted under controlled potential conditions. However, the anodic potential is not well controlled when DMFCs are operating. Therefore, the observed HCHO burst is likely to better represent the actual scenario when a DMFC device is switching between operating (with potential) and resting (open circuit) conditions. From a technical point of view, *in situ* determination of HCHO has been standing as a technical challenge for a long time. The present work demonstrates the capability for quantitative and label-free visualizing of HCHO production based on its refractive index. The sufficient temporal and spatial resolutions of SPRi thus promise the possibility to clarify the debated mechanism underneath the production of HCHO in methanol oxidation reactions.

## Conflicts of interest

There are no conflicts to declare.

## Supplementary Material

Supplementary movie

Supplementary information
