# Supplementary material for: In operando imaging of self-catalyzed formaldehyde burst in methanol oxidation reactions under open circuit conditions
Source: Chem Sci. 2018 Feb 26;9(13):3318–23. doi: 10.1039/c7sc05347a (PMC5932601; doi:10.1039/c7sc05347a)
Supplement: Supplementary file 2 [file SC-009-C7SC05347A-s002.pdf]

Electronic Supplementary Material (ESI) for Chemical Science.
This journal is © The Royal Society of Chemistry 2018

Electronic Supplementary Material (ESI)

In operando imaging of self-catalyzed formaldehyde burst in methanol oxidation under open circuit conditions

Liang Yuan, Meng Li, Tinglian Yuan, Yimin Fang, Wei Wang*

State Key Laboratory of Analytical Chemistry for Life Science, School of Chemistry and Chemical Engineering, Nanjing University, Nanjing 210023, China

*Corresponding Author: Wei Wang
Email: wei.wang@nju.edu.cn

1 Experimental Procedures
1.1 Preparation and characterization of PtNPs
1.2 SPR imaging (SPRi) and image processing
1.3 Spectrophotometric assay of HCHO

2 Results and Discussion
2.1 Electrocatalytic activity of the Pt-disk chip for methanol electro-oxidation
2.2 3-Dimensional semi-infinite diffusion model
2.3 Reaction mechanism of MB-bromate system for formaldehyde detection
2.4 The refractive index of formaldehyde and methanol
2.5 The electrochemical current and SPR intensity in the CA measurement

3 Supporting figures
Fig. S1 TEM, SEM and DLS characterization of PtNPs
Fig. S2 The cyclic voltammetry profiles of Pt-disk chip in different electrolytes
Fig. S3 Enlarged view of SPR intensity of Pt-disk chip under different potentials
Fig. S4 Detection of HCHO via MB-$KBrO_3$ system
Fig. S5 A ring-shape ROI surrounding the Pt-disk
Fig. S6 Refractive index changes of CH_3OH, HCHO and H_2SO_4
Fig. S7 Time evolution of the OCP at the Pt-disk chip in 0.5 M H_2SO_4 solution
Fig. S8 The current of the Pt-disk chip in chronoamperometry measurement

4 Supporting movie
Movie S1 A wave-like burst of produced HCHO occurs surrounding the Pt-disk.

5 References

1 Experimental Procedures

1.1 Preparation and characterization of PtNPs

The platinum nanoparticles (PtNPs) are synthesized through a rapid and one-step method according to previous literature.[1] Briefly, the PtNPs are prepared by mixing H_2PtCl_6 (3 mL, 18.9 mM), ascorbic acid (0.2 mL, 1 M) and NaOH (15 μL, 5 M) together in a beaker which contains 18 mL H_2O. After heating the mixture in a water-bath at 60 ^oC for 10 min without any disturbance, the brown suspension containing monodisperse PtNPs are obtained. Such surfactant-free PtNPs possess definitely "clean" surfaces and can be directly used for subsequent experiments after simple washing with water. The morphology and structure of the as-synthesized sample are characterized by TEM (JEM-2100) and SEM (Shimadzu, S4800). Fig. S1 shows that the PtNPs are almost monodisperse with a mean particle size of 500 nm, which is further confirmed by the dynamic light scattering (DLS, Malvern Nano-ZS90 Zetasizer) data. Additionally, the high-resolution TEM image shows that the PtNPs are in the polycrystalline state, of which the *d* spacing is 0.22 nm and 0.19 nm, which can be assigned to be [111] and [200] planes of Pt, respectively.

1.2 SPR imaging (SPRi) and image processing

The prism-based SPRi setup is comprised of an LED light source, collimating lens, prism, polarizer and a high-speed charge-coupled device (CCD) camera (Pike, Allied Vision Technologies). Specifically, the *p*-polarized red light from the LED (wavelength 670 nm) is collimated and directed to the sensor chip via a triangular prism at an incident angle near SPR resonance angle. The SPR sensor chip is a BK7 glass cover slide coated with 2 nm chromium followed by 47 nm gold, which is also used as the working electrode.

Before use, each chip is washed with water and ethanol, followed by briefly annealing with a hydrogen flame to remove surface contamination. 0.2 μL of the PtNPs solution is dropped onto the chip surface via the pipette, followed by dried in a vacuum. Then this Au chip, used as the SPR sensing surface, is put upon the prism using index-matching fluid between them. A PDMS chamber (Flexiperm micro12, Sarstedt) is placed on top of the gold surface for housing the reaction solution and electrodes. As to electrochemical system, both the counter electrode (CE) and reference electrode (RE) are inserted to the electrochemical cell from the top opening. SPRi images are recorded at a speed of 7.6 frames per second (fps). Then the intensity is determined by calculating the average intensity within a fixed small rectangle region-of-interest (ROI) selected close to the Pt-disk.

1.3 Spectrophotometric assay of HCHO

Determination of formaldehyde is accomplished by a spectrophotometric method with a methylene blue-bromate ($MB-KBrO_3$) system. All solution is kept in a 30 ℃ thermostat water batch for 20 min before initiation of the reaction. To a series of 5 mL tubes, 40 μL of 0.05 mM MB solution, 0.6 mL of 0.5 M H_2SO_4 (or electrolyte after electro-oxidation), and different amounts of HCHO are added. Then 100 μL of 10 mM bromate solution is added and the solution is diluted to 1.8 mL with deionized water.

After shaking, the mixed solution is transferred into quartz cell for subsequent measurement by a spectrophotometer (Cary 100, Agilent Technologies). The variation in absorbance of MB (λ_{max} = 664 nm) versus time is continuously monitored after the addition of bromate solution. The blank reaction is performed according to the same procedure without addition of HCHO and the absorbance is labeled as A_0. The standard curve which the difference of absorbance between blank and sample in 664 nm (A_0 - A) of MB versus the concentration of HCHO is also constructed.

2 Results and Discussion

2.1 Electrocatalytic activity of the Pt-disk chip for methanol electro-oxidation

A CHI 760E electrochemical workstation (Shanghai Chenhua Instrumental CO., Ltd., China) is employed for all the electrochemical experiments with a Pt wire and Ag/AgCl served as the counter electrode (CE) and the reference electrode (RE), respectively. The SPR sensor chip is also used as the working electrode (WE).

Pre-synthesized colloids containing 500 nm spherical Pt nanoparticles are deposited on gold-coated coverslip to form a porous and disk-like Pt-catalyst (Pt-disk) with a diameter of ~1000 microns. Gold film acts as both the working electrode and the optical interface to excite surface plasmon polaritons. Electrochemical characterization in the presence of methanol displays a pair of well-documented oxidative current peaks in the cyclic voltammogram (Fig. S2, red line), demonstrating that as-obtained Pt-disk can efficiently catalyze the electrochemical oxidation of methanol. Gold substrate does not interfere with the methanol oxidation under the present conditions, as indicated by the background cyclic voltammogram curve in the absence of Pt-disk (Fig. S2, blue line).

Fig. S2 shows the electrochemical current results of the voltammetry measurements made on Pt-disk chip without and with the methanol in acidic electrolyte solution (0.5 M H_2SO_4) at ambient temperature. The electrocatalytic activities of this Pt-disk chip are investigated by traditional cyclic voltammetry profiles at a sweep rate of 50 mV/s from 0 V to 0.8 V (vs NHE). As shown in Fig. S2 (black line), the Pt-dot chip produces several peaks similar to that of polycrystalline Pt electrode in 0.5 M H_2SO_4 solution. Typically, the peak corresponding to the formation of platinum oxide appears after 0.6 V, and another peak that corresponding to the reduction appears at 0.48 V. However, when the electrolyte contains the methanol, the significant redox peaks of methanol oxidation appears at 0.56 V in the positive-going scan and 0.45 V in the negative-going scan, respectively, demonstrating the reliability of such Pt nanoparticles covered Au chip as working electrode in the electrocatalysis of methanol (Fig. S2, red line). The as-prepared Pt-disk chip exhibits the equivalent catalytic performance due to the relatively lower anodic overpotential.

2.2 3-Dimensional semi-infinite diffusion model

According to the diffusion model, we assume an immediate (t = 0) and point (x = 0, y = 0, z = 0) release of M molecules. These molecules diffuse at a semi-infinite space ($-\infty < x < +\infty$, $-\infty < y < +\infty$, $0 \leq z < +\infty$). Diffusion coefficient (D) is homogenous at any

space location. Then, the basic equation is:

$$C(x,y,z,t) = \frac{M}{(\sqrt{4\pi t})^3\sqrt{D_xD_yD_z}}\exp\left(-\frac{x^2}{4D_xt}-\frac{y^2}{4D_yt}\right)\times\left[\exp\left(-\frac{(z-H)^2}{4D_zt}\right)+\exp\left(-\frac{(z+ [illegible]}{4D [illegible]}\right.\right.$$

where H is the height of source point, and H = 0 here. Normally SPR measures the concentration distribution in the basal plane of this semi-infinite diffusion space. Penetration depth of surface plasmon wave (200 nm) is significantly smaller than the diffusion distance (r), which is often in the scale of 100 microns. So, we actually measure the concentration distribution in the basal plane (z = 0). If we solely focus on the basal plane and let distance $r = \sqrt{x^2 + y^2}$, then

$$C(r,t) = \frac{M}{(\sqrt{4\pi Dt})^3}\times exp\left(-\frac{r^2}{4Dt}\right)\times 2$$

At each location, the initial concentration ($C(r,0) = 0$) is zero. Subsequently, it will increase due to the species diffused from the source. Eventually the concentration would decrease and approach zero again. The time required to reach the maximal concentration is a function of distance (r) to the center of the Pt-disk. It takes shorter time to reach the maximal concentration for a point with shorter distance. The time is defined by the first order derivative of dC/dt.

$$\frac{dC(r,t)}{dt} = \frac{d}{dt}\left(\frac{M}{(\sqrt{4\pi Dt})^3}\times exp\left(-\frac{r^2}{4Dt}\times 2\right)\right)$$

$$= \frac{2M}{(\sqrt{4\pi D})^3}\times t^{-5/2}\times exp\left(-\frac{r^2}{4Dt}\right)\times\left[-\frac{3}{2}+\frac{r^2}{4Dt}\right]$$

= 0

Therefore, $\left[-\frac{3}{2}+\frac{r^2}{4Dt}\right] = 0$, we further get the equation that $t = \frac{r^2}{6D}$ or $r = \sqrt{6D}\times t^{1/2}$. Together with the experiment data, the results follow the relationship that $L = \sqrt{6D}\times t^{1/2}$, in which the slope ($k = \sqrt{6D}$) of this linear fitting equation is 1.7×10^{-4}. Thus, the diffusion coefficient D is 4.8×10^{-9} m^2/s.

2.3 Reaction mechanism of MB-bromate system for formaldehyde detection

Methylene blue (MB) is a dye that can be oxidized with strong oxidizing agents at slow reaction. Formaldehyde can accelerate the reacting rate at ultra-trace level, and the catalytic effect increased with increasing concentration of the formaldehyde.[2-5] The mechanism of the reaction between MB and potassium bromate may attributed to the equations as below:

(a) Absence of HCHO

$BrO_3^- + H^+ + MB (Red) \rightarrow Br^- + MB (Ox)$ (S-1)

Slow

(b) Presence of HCHO

$BrO_3^- + H^+ + HCHO \rightarrow Br^- + CO_2 + H_2O$ (S-2)

Fast

$BrO_3^- + H^+ + Br^- \rightarrow Br_2 + H_2O$ (S-3)

Fast

$Br_2 + MB (Red) \rightarrow Br- + MB (Ox)$ (S-4)

Fast

"Red" represents the reduced form of MB, and "Ox" is the oxidized form. Generally, the reaction [S-Eq. (1)] is very slow in the absence of formaldehyde, so the bromine ion (BrO_3^-) oxidizes MB to produce only weak decrease of the absorbance of dye. When trace amount of formaldehyde is added into the system, however, the BrO_3^- in reaction [S-Eq. (2)] liberates the bromine ion (Br^-) very fast and further reacts with bromate quickly to generate Br_2 in situ in the system. Such fresh generated Br_2 is a nascent oxidant and can catalyze the MB (Red) being quickly oxidized to MB (Ox) in reaction [S-Eq. (4)]. This reaction is monitored spectrophotometrically by measuring the decrease in absorbance of MB at about 664 nm, which can be used to measure the concentration of formaldehyde.

Thus, we take this method to further identify the formaldehyde produced in methanol oxidation, and the absorbance spectra of different solutions by UV-vis spectrometry (Cary 100, Agilent Technologies) are displayed in Fig. S4 for checking its reliability. The reaction rate of the MB with $KBrO_3$ in sulfuric acid is very slow and the absorbance at about 664 nm is still strong (Fig. S4-a, curve 1). However, the rate of MB oxidized by $KBrO_3$ increases quickly in the presence of HCHO (curve 4) and the peak absorbance decreases in large range comparing with the curve 1. We note that no pronounced catalytic effect can be find in the CH_3OH or HCOOH system. The kinetic curves for the absorbance variation as a function of time can give a better illustration and it is demonstrated that MB could be quickly oxidized by bromate ion in the presence of both determinate HCHO solution and real electro-oxidation sample (Fig. S4-b). Thus, it is reliable to use this selective method to detect the real sample of methanol oxidation without having to consider the interference from the reactant methanol and intermediate formic acid.

After electro-oxidation of the H_2SO_4-CH_3OH solution at different constant potentials for 30 s on the Pt-dot chip, the electrolytes are collected and then sampled with MB-$KBrO_3$ solution for subsequent online analysis by UV-vis spectroscopy. We also collect the different absorbencies of freshly prepared standard solutions in the range of 0.3-100 μM. The linear calibration plot in Fig. S4-c demonstrates the assay sensitivity and precision. The limit of detection as near 200 nM. As a result, the produced HCHO quantity under different applied pre-oxidation potentials is determined from the absorbance change in comparison to the calibration curve.

2.4 The refractive index of formaldehyde and methanol.

We have the SPR intensity change when adding different amount of HCHO and CH_3OH into the 0.5 M H_2SO_4 +1.0 M CH_3OH electrolyte (EL-1 solution), respectively.

The corresponding refractive index (RI) of HCHO and CH_3OH solutions are also investigated at the same time. The RI of single pure formaldehyde solution (37%, v/v) is measured as 1.377 at the temperature of 15 ℃ by WAY-2S ABBE Digital Refractometer (Shanghai INESA Physico-Optical Instrument CO., Ltd, China). As to methanol solution (99.9%, v/v), its RI value is 1.331. When we add CH_3OH and HCHO into the 0.5 M H_2SO_4 +1.0 M CH_3OH electrolyte (EL-1) solution, respectively, the RI of mixed solution gradual increases with the increasing concentration (Fig. S6). And addition of HCHO has higher rising rate, which is consistent with the conclusion of SPR intensity variation in Fig. 2d. Thus, SPRi can easily catch the emergence of HCHO, as well as the concentration change of HCHO, due to the different RI.

2.5 The electrochemical current and SPR intensity in the CA measurement.

The electrochemical current curves in the stage 4 of CA measurement are also recorded and displayed in Fig. S8. We find that the current value in H_2SO_4-CH_3OH solution is always larger than that in corresponding separated H_2SO_4 solution. And the current values of Pt-dot in H_2SO_4-CH_3OH subtracted by that in H_2SO_4 are all positive values, which can be viewed as oxidation currents, indicating the generation of formation in this stage 4 actually derives from the oxidation of methanol indeed. If we integrate the differential current over time, then we will get the total amount of electric charge inside the stage 4.

As to the SPR intensity, the low-potential of 0.4 V is an exception and discussed in Fig. 3b. For the low-potential from 0 V to 0.3V, the amount of generated formaldehyde has a minimum at about 0 V, which is close to then OCP of system (-0.05 V). The more far away the OCP, the more electrons transfer at the chip/electrolyte interface and contribute to oxidize the methanol to formaldehyde. In other words, under the premise of adsorbing same amount of Pt oxides when the applied low-potential is very close to OCP, it only needs less driving force (i.e. electrons) to return to the system steady state and the production of formaldehyde decreases accordingly. However, it's important to point out that too negative cathode potential (e.g. -0.2 V) was unfavorable to the oxidation of methanol molecule, and no obvious SPR intensity peak appeared.

3 Supporting figures

Fig. S1 TEM, SEM and DLS characterization of PtNPs.

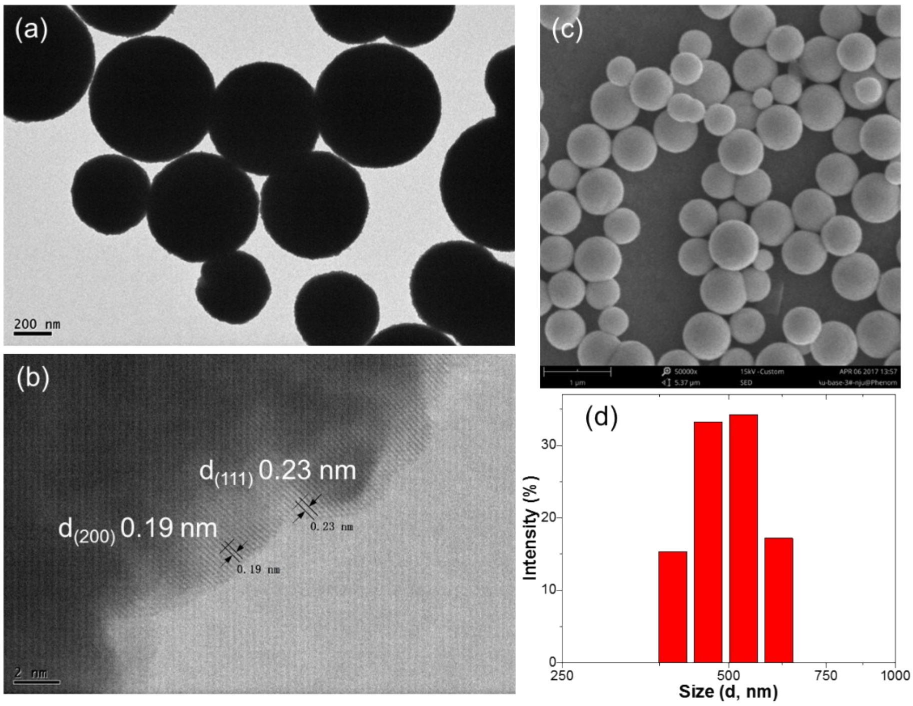

Fig. S1 (a) TEM, (b) HRTEM, and (c) SEM images of the synthesized single Pt nanoparticles (PtNPs). (d) Dynamic light scattering plot of the obtained PtNPs. The measured particle size is around 500 nm.

Fig. S2 The cyclic voltammetry profiles of Pt-disk chip in different electrolytes

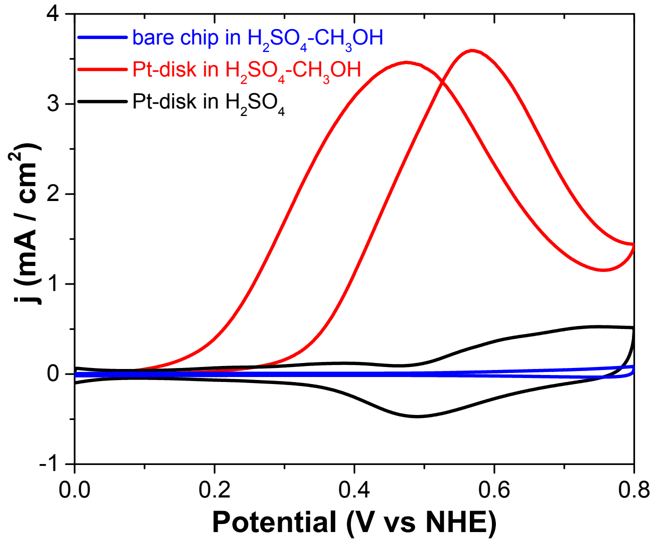

Fig. S2 Comparison of the cyclic voltammetry profiles of Pt-disk chip in 0.5 M H_2SO_4 + 1.0 M CH_3OH electrolyte (red line) and only 0.5 M H_2SO_4 electrolyte (black line), as well as the bare Au chip without Pt-disk in 0.5 M H_2SO_4 + 1.0 M CH_3OH electrolyte (blue line). Scan rate is 50 mV/s, and the potential range is from 0 V to 0.8 V.

Fig. S3 Enlarged image of SPR intensity of the Pt-disk chip undergoes different potentials

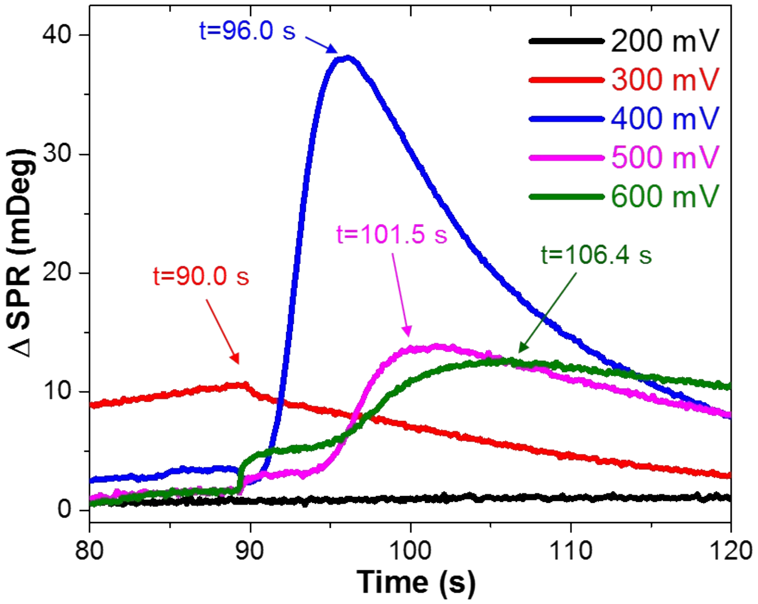

Fig. S3 An enlarged image of the SPR intensity of the Pt-disk chip that undergoes different pre-oxidation potentials.

Fig. S4 Detection of HCHO via MB-$KBrO_3$ system

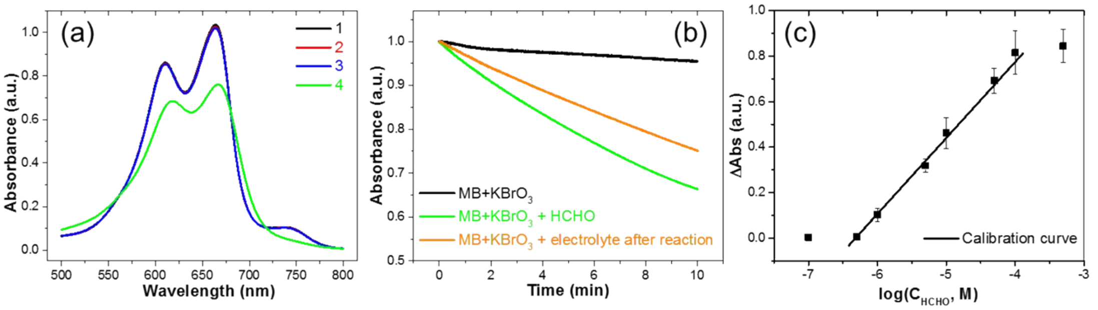

Fig. S4 (a) The absorption spectra of methylene blue (MB) in reaction system (1) H_2SO_4 + $KBrO_3$ + MB; (2) H_2SO_4 + $KBrO_3$ + MB + HCOOH; (3) H_2SO_4 + $KBrO_3$ + MB + CH_3OH; (4) H_2SO_4 + $KBrO_3$ + MB + HCHO. Condition: MB 6.7×10^{-5} M; $KBrO_3$ 0.01 M; H_2SO_4 0.5 M; temperature 30 ^{o}C; reaction time 2 min. (b) Kinetic curves for the absorbance change at 664 nm as a function of time in different solutions including H_2SO_4 + $KBrO_3$ + MB, H_2SO_4 + $KBrO_3$ + MB + HCHO, and H_2SO_4 + $KBrO_3$ + MB + electrolyte after electro-oxidation of methanol. (c) The calibration curve of UV absorbance changes at 664 nm versus different HCHO concentrations. In order to simulate actual reaction condition, the MB reaction system here is composed of 0.5 M H_2SO_4, 1.0 M CH_3OH, 6.7×10^{-5} M MB, 0.01 M $KBrO_3$, and appointed standard HCHO solution.

Fig. S5 A ring-shape ROI surrounding the Pt-disk

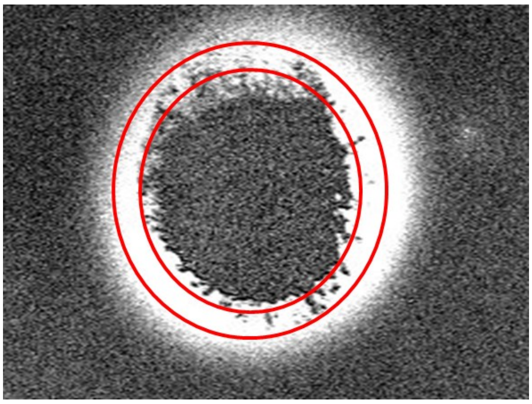

Fig. S5 The illustration of a ring-shape region-of-interest (ROI) surrounding the Pt-disk (red circle) for the determination the SPR intensity in order to avoid the deviation or interference caused by inhomogeneous generation and diffusion of HCHO.

Fig. S6 Refractive index changes of CH_3OH, HCHO and H_2SO_4

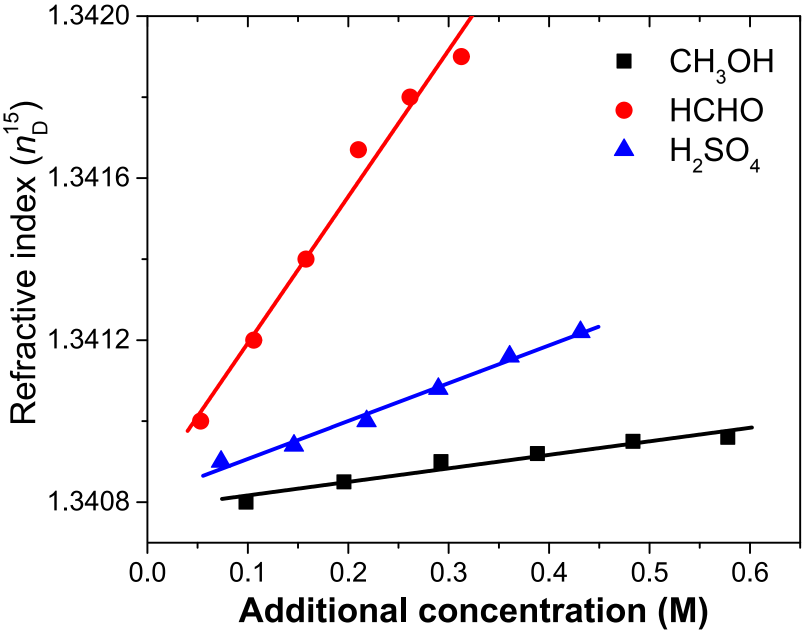

Fig. S6 The refractive index changes of mixed electrolyte solutions as a function of additional concentration of added CH_3OH, HCHO and H_2SO_4.

Fig. S7 Time evolution of the OCP at the Pt-disk chip in 0.5 M H_2SO_4 solution

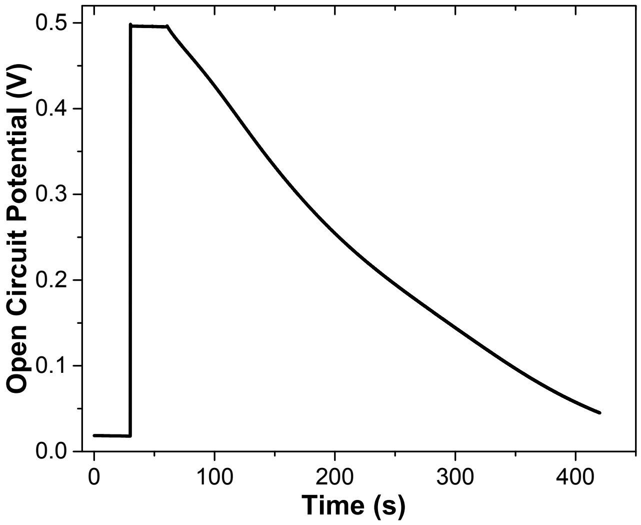

Fig. S7 Time evolution of the open circuit potential at the Pt-disk chip in 0.5 M H_2SO_4 electrolyte undergoes the process of firstly in the absence of external potential for 30 s, then applying the pre-oxidation potential of 0.5 V for another 30 s, subsequently switching off the applying potential control together with recording the OCP variation.

Fig. S8 The current of the Pt-disk chip in chronoamperometry measurement

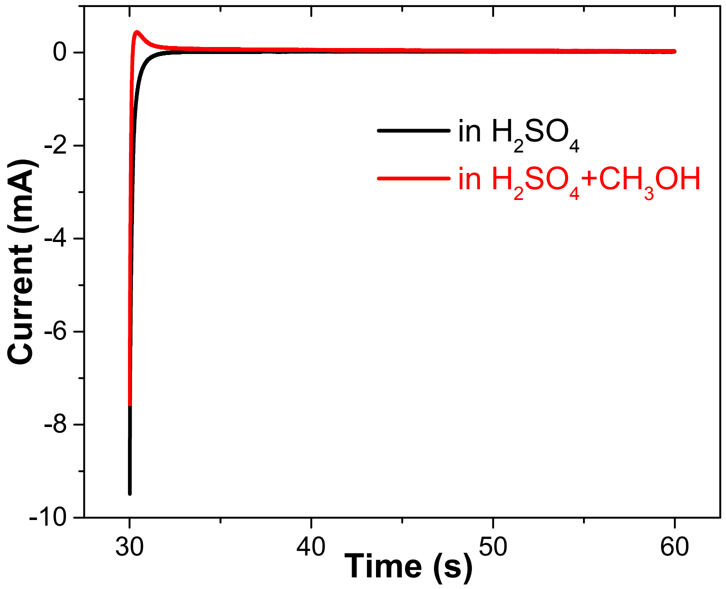

Fig. S8 The corresponding current curves of the Pt-disk chip in chronoamperometry measurement when the applied potential is stepped from 0.5 V to and hold at lower potential of 0 V. The red line is the current from the H_2SO_4 + CH_3OH solution, while the black line is from the H_2SO_4 solution.

4 Supporting movie

Movie S1: A wave-like burst of generated HCHO diffusion occurs surrounding the Pt-disk after the withdrawal of pre-oxidation potential. Different ROIs adjacent to the Pt-disk show different SPR intensity variation.

5 Reference

1. J. Wang, X. B. Zhang, Z. L. Wang, L. M. Wang, W. Xing and X. Liu, *Nanoscale*, 2012, **4**, 1549-1552.
2. M. R. Shishehbore, N. Nasirizadeh and A. A. Kerdegari, *Anal. Sci.*, 2005, **21**, 1213-1216.
3. X. J. Cui, G. Z. Fang, L. Q. Jiang and S. Wang, *Anal. Chim. Acta*, 2007, **590**, 253-259.
4. J. Z. Li, P. K. Dasgupta and W. Luke, *Anal. Chim. Acta*, 2005, **531**, 51-68.
5. Y. F. Tang, H. Chen, C. Weng, X. H. Tang, M. L. Zhang, Q. Q. Yang, T. Hu and C. Q. Cai, *Spectrochim. Acta, Part A*, 2014, **125**, 126-130.
